# Abnormal epithelial homeostasis in the cornea of mice with a destrin deletion

**Published:** 2008-10-28

**Authors:** W. Zhang, J. Zhao, L. Chen, M. M. Urbanowicz, T. Nagasaki

**Affiliations:** 1 Department of Ophthalmology, Columbia University, New York, New York; 2 Department of Ophthalmology, the Second Affiliated Hospital of China Medical University, Shenyang, China

## Abstract

**Purpose:**

*Dstn^corn1^* mice lack normal destrin expression and develop corneal abnormality shortly after birth such as epithelial hyperplasia and total vascularization. Thus, the mice serve as a model for ocular surface disorders. To determine the nature of epithelial defects, we examined whether epithelial homeostasis is altered in these corneas.

**Methods:**

*Dstn^corn1^* mice were crossed with ubiquitous GFP mice to generate a double homozygous line, GFP-*Dstn^corn1^*, and cell movements were determined by whole-mount histology and in vivo time-lapse microscopy, tracking the change of epithelial GFP patterns. Rates of cell division and the presence of label-retaining cells (LRCs) were determined by systemic bromodeoxyuridine (BrdU). Epithelial expression of keratins 8, 12, and 15, and MUC5AC were determined by whole-mount immunofluorescence.

**Results:**

Epithelial cells in an adult GFP-*Dstn^corn1^* cornea were generally immobile with no sign of directed movement for the entire life of the animal. These cells were not senescent because more than 70% of basal epithelial cells incorporated BrdU over a 24 h period. LRCs were widely distributed throughout a GFP-*Dstn^corn1^* cornea. The epithelium of a GFP-*Dstn^corn1^* cornea contained a mixed population of cells with a corneal and a conjunctival phenotype as judged by the expression of keratins and MUC5AC.

**Conclusions:**

Epithelial cells of an adult GFP-*Dstn^corn1^* cornea are generally stationary, mitotically active, and contain LRCs, indicating that the epithelium is self-sustained, which in turn suggests that epithelial stem cells are present within the cornea. Epithelial homeostasis of adult GFP-*Dstn^corn1^* corneas is abnormal, mimicking that of a normal conjunctiva or a pathological, conjunctivalized cornea.

## Introduction

*Dstn^corn1^* mice are a spontaneous mutant line that exhibit ocular surface abnormalities shortly after birth, including epithelial hyperplasia and stromal vascularization [1]. The molecular defect has been identified as deficiency of destrin [2], an actin binding protein also known as actin depolymerizing factor (ADF). It is a member of the actin severing proteins that share sequence and functional similarity with cofilins [3]. In addition to the naturally occurring *Dstn^corn1^* mice, destrin-knockout mice generated by gene targeting also exhibited mild corneal hyperplasia [4]. The requirement of destrin seems to be unique to the cornea because *Dstn^corn1^* mice are fertile and appear normal, apart from the corneal defect. However, it is not known how a destrin mutation leads to phenotypic changes in a *Dstn^corn1^* cornea.

Histology of *Dstn^corn1^* corneas showed that the hyperplastic epithelium expressed an increased level of keratin 14 and involucrin while the level of keratin 12 was not altered [5]. It has been shown that hemangiogenesis and lymphangiogenesis in the *Dstn^corn1^* cornea depend on vascular endothelial growth factor receptor 3 (VEGFR3) signaling [6], but its relationship with a destrin mutation is not known. Recently it was suggested that vascularization of *Dstn^corn1^* corneas arises from lack of soluble VEGF receptor, sflt-1, which was proposed as an essential factor for maintenance of avascularity in a normal cornea [7]. In this scenario, the loss of destrin would be the initial event that leads to the loss of sflt-1 in a *Dstn^corn1^* cornea, but this causal relationship has not been established at a molecular level.

Two of the major defects in a *Dstn^corn1^* cornea, vascularization and abnormal epithelium, may arise independently of each other as a consequence of destrin deficiency. Alternatively, there may be a causal relationship between the two, i.e., either one may be a prerequisite for the other. Gross and histological observations suggested that a corneal surface irregularity occurred about a week before signs of neovascularization [1], favoring an idea that epithelial defects may trigger neovascularization. However, initial molecular events leading to vascularization may have been undetectable using histological techniques, and therefore, other possibilities remain viable at this time.

In this study, we focused on dynamic aspects of epithelial defects in a *Dstn^corn1^* cornea by studying its epithelial homeostasis in the hope that this knowledge will eventually help the molecular elucidation of corneal defects due to destrin mutations. Our results suggest that epithelial homeostasis of a *Dstn^corn1^* cornea is abnormal and similar to that of a normal conjunctival epithelium or a pathological epithelium of a conjunctivalized cornea, both of which feature an underlying stromal vasculature.

## Methods

### Animals

The animal studies adhered to the Association for Research in Vision and Ophthalmology Statement for the Use of Animals in Ophthalmic and Vision Research and was approved by the institutional animal care and use committee. *Dstn^corn1^* (Stock Number 001649) and CAG-EGFP (Stock Number 003115) mice were obtained from the Jackson Laboratory (Bar Harbor, ME), crossed, and bred to generate a strain with homozygosity to both traits, which we refer to as GFP-*Dstn^corn1^* in this paper.

### Histology

For histology, the eyes were isolated after sacrificing the animal with intraperitoneal pentobarbital (100 mg/kg). Whole-mounts containing an entire area of ocular surface were prepared as described previously [8] and stained with DAPI to reveal nuclei. Fluorescence patterns of DAPI and GFP were visualized with a fluorescence microscope with appropriate filter sets (Axioskop2, Carl Zeiss, Oberkochen, Germany). Overlapping microscopic images were acquired digitally (Orca 100; Hamamatsu, Hamamatsu City, Japan and Metamorph; Molecular Dynamics., Downingtown, PA), and assembled with Photoshop (Adobe Systems, San Jose, CA) to prepare a large image file of the entire ocular surface for analysis.

Immunohistochemistry was performed with cornea whole mounts that were fixed with 1% paraformaldehyde in PBS for 30 min. For BrdU staining, corneal whole-mounts were pre-treated for epitope retrieval with 2 N HCl for 15 min at 37 °C. *Dstn^corn1^* corneas were further processed by incubation with 0.2 mg/ml pepsin (Sigma-Aldrich, St. Louis, MO) in 0.1 N HCl for 5 min at 37 °C so that antibody penetration to basal layers was satisfactory. For double immunofluorescence staining, corneal GFP fluorescence was quenched completely by treatment with methanol before antibody incubation. Rat anti-BrdU antibody was from Serotec (Raleigh, NC). Rabbit anti-destrin antibody (GV-13) was purchased from Sigma-Aldrich. Rat anti-K8 antibody (Troma-I) was obtained from Developmental Studies Hybridoma Bank (University of Iowa, Iowa City, IA). Goat anti-K12 (L15) and goat anti-MUC5AC (K20) were from Santa Cruz Biotechnology (Santa Cruz, CA). Mouse anti-K15 was from Lab Vision (Fremont, CA) and rabbit anti-K15 was from ProteinTech (Chicago, IL). Secondary antibodies conjugated with either Texas-red, Cy2, or Cy3 were purchased from Jackson Immunoresearch Laboratories (West Grove, PA). All immunostaining reactions without primary antibodies were negative under the same image acquisition conditions for staining with primary antibodies. This ensured that fluorescent signals were due to specific reactions of primary antibodies to target tissues.

### In vivo microscopy

In vivo microscopy and digital imaging were performed as described previously [8,9] using an M2Bio fluorescence microscope system (Kramer Scientific, Valley Cottage, NY), which is based on an SV11 stereo microscope (Carl Zeiss). Mice were anesthetized with a stream of 3% isoflurane in oxygen. The eye was lightly proptosed with a thin vinyl-coated, U-shaped flexible metal wire for microscopy. GFP fluorescence images were obtained with a 1.6X objective with a zoom at 1.0X using a digital camera (Coolsnap ES; Photometrics, Tucson, AZ) controlled by MetaVue (Molecular Devices, Downingtown, PA). Image resolution was approximately 4.0 µm/pixel under these conditions. At each time point, at least seven images were taken, three at the central cornea at different focal planes, and one each at the superior, the inferior, the temporal, and the nasal portion of the peripheral cornea. This was facilitated by a set of custom-made gimbals, which accommodated a mouse platform and could be rotated freely to bring an ocular surface area of interest to the apex to face the objective. GFP patterns were recorded at intervals of one to four weeks, and movement of GFP positive cells was analyzed from time lapse sequences as before [8,10].

### Labeling of DNA with BrdU

To determine cell division rates, DNA was metabolically labeled with bromodeoxyuridine (BrdU; Sigma-Aldrich) for 24 h in live mice of 13-20 weeks. BrdU (0.6 mg/day) was given systemically with an Alzet osmotic pump (model 1003D; DURECT Corp., Cupertino, CA) that was implanted subcutaneously in the back. The animal was sacrificed 24 h later to determine DNA synthesis during the 24 h period. Whole mounts were prepared to determine BrdU-incorporating cells by immunohistochemistry (see above). BrdU-labeling rates over 24 h were determined as a ratio of the number of BrdU positive cells and the number of DAPI-stained cells in an area of 170×210 µm in digitized microscopic images. At least six such areas were selected randomly in each of the five eyes for GFP-*Dstn^corn1^* corneas and three eyes for CAG-EGFP corneas.

For determination of label-retaining cells (LRCs), four adult mice (18-24 weeks old) were labeled with BrdU with a subcutaneous Alzet pump (model 2002) for two consecutive weeks. The Alzet pump was removed at the end of the two-week labeling, and the mouse was sacrificed eight weeks later to harvest both eyes when only slow cycling cells would retain BrdU in their nuclei. After whole mount BrdU immunofluorescence, individual BrdU retaining cells (i.e., LRCs) were located under a fluorescence microscope and plotted manually on a print-out of a whole mount outline. Manual determination of BrdU positive cells under the microscope was aided by focusing up and down and switching between BrdU and DAPI channels to ensure only epithelial BrdU signals were counted.

## Results

### Generation of GFP-*Dstn^corn1^* hybrid mice

**Figure 1:**
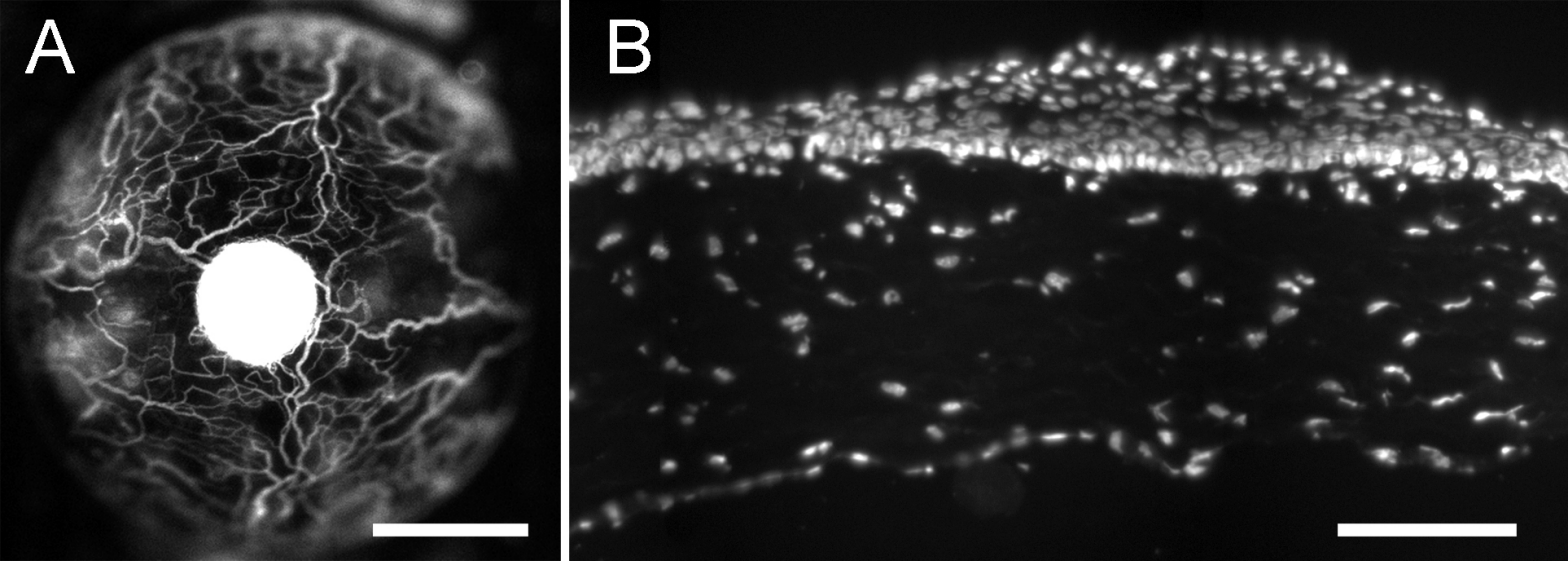
Expression of *Dstn^corn1^* phenotype by GFP-*Dstn^corn1^* corneas. **A**: The corneal vasculature was visualized by sulforhodamine-101 angiography with a 15-week-old GFP-*Dstn^corn1^* mouse. **B**: A cryosection of a cornea from a 12-week-old GFP-*Dstn^corn1^* mouse was stained with DAPI, showing epithelial hyperplasia. Bar: 1 mm (**A**), 100 µm (**B**).

To observe homeostatic movement of epithelial cells, we crossed CAG-EGFP mice with *Dstn^corn1^* mice to generate a line, GFP-*Dstn^corn1^*, which was homozygous to both traits. Double homozygosity was confirmed by the respective phenotype. For *Dstn^corn1^* phenotype, spontaneous corneal vascularization (Figure 1A) and epithelial hyperplasia (Figure 1B) were observed as with *Dstn^corn1^* homozygous mice [1]. For GFP phenotype, fluorescence intensity of the corneal epithelial GFP indicated homozygosity [10,11]. We examined more than 100 GFP-*Dstn^corn1^* mice, which all exhibited spontaneous corneal vascularization and epithelial hyperplasia without exception. Destrin, which is lacking in *Dstn^corn1^* mice [2], was absent in GFP-*Dstn^corn1^* corneas while it was uniformly positive in CAG-EGFP corneas as judged by destrin whole mount immunofluorescence (data not shown). These results indicated that a GFP-*Dstn^corn1^* cornea was functionally equivalent to a *Dstn^corn1^* cornea. Accordingly, all of the following experiments were performed with GFP-*Dstn^corn1^* mice.

### Cell movement in a GFP-*Dstn^corn1^* cornea

**Figure 2:**
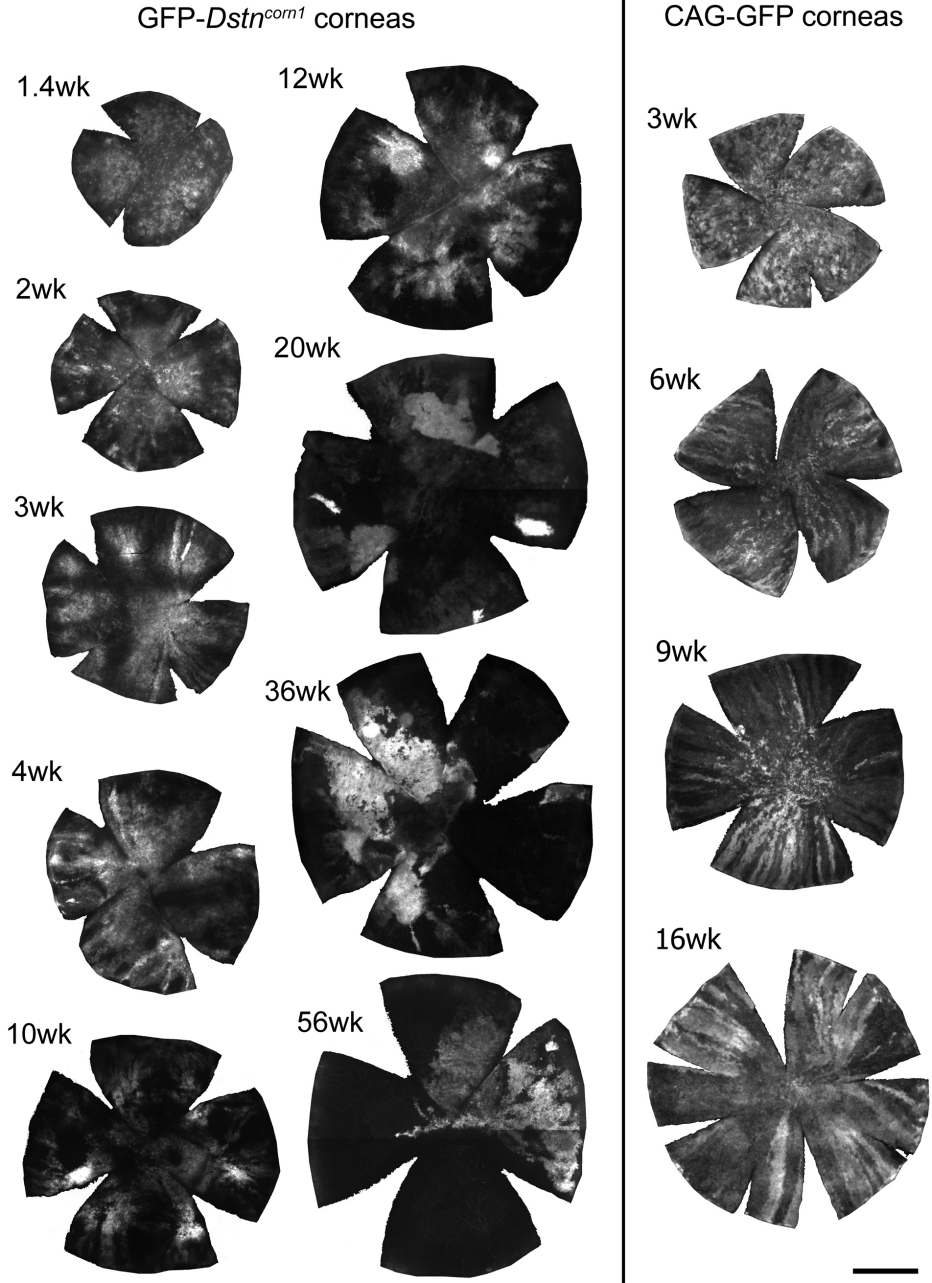
Developmental changes of GFP patterns in GFP-*Dstn^corn1^* corneas and normal CAG-EGFP corneas. Flat whole mount corneas were prepared from respective mice of indicated ages. Cuts were made to mount the cornea flat. GFP patterns were imaged and digitally recorded. Only the cornea area is shown. Radial GFP stripes appeared in normal CAG-EGFP corneas around four weeks and remained in all corneas of all ages we observed (see also [10]). GFP stripes were detected in young GFP-*Dstn^corn1^* mice at three to six weeks, but there were none in adult corneas, which instead showed globular and diffuse GFP patterns. Bar: 1 mm.

To study epithelial homeostasis in a GFP-*Dstn^corn1^* cornea, we first analyzed homeostatic, natural movements of epithelial cells by histology and in vivo time lapse microscopy. In normal CAG-EGFP mice (Figure 2, right panel), corneal GFP patterns were mosaic in two-week-old animals (a three-week cornea is shown in Figure 2) while radial stripes emerged at the limbus at three to four weeks and continued to move centripetally and reached the center by 10 weeks [10]. GFP-*Dstn^corn1^* corneas (Figure 2, left panel) also exhibited mosaic patterns after birth, but radial stripes appeared as early as two weeks at the peripheral cornea. The centripetal progression of budding radial stripes stopped at four to six weeks and never reached the center of the cornea. Instead, GFP patterns became diffuse and globular without a sign of extended stripes, radial or otherwise, indicating that there was neither centripetal movement nor sustained directional movement of epithelial cells in an adult GFP-*Dstn^corn1^* cornea.

**Figure 3:**
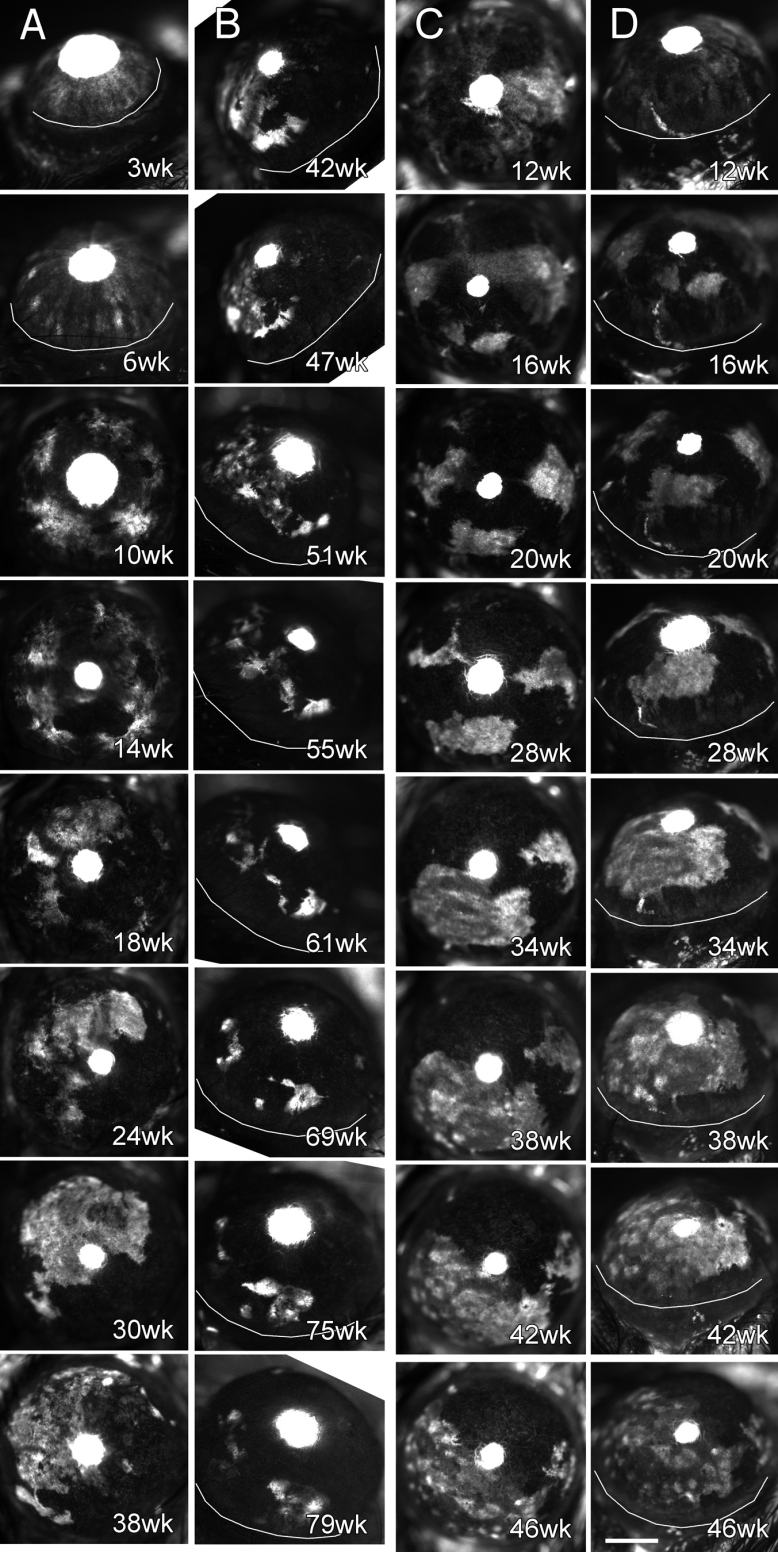
Two representative time lapse sequences of corneal GFP in GFP-*Dstn^corn1^* mice. One representative time lapse is shown **A** and **B** and the other in **C** and **D**. Corneal GFP patterns were imaged with in vivo fluorescence microscopy at an interval of one to four weeks, and selected images are shown together with the age of the mouse. White areas represent GFP positive epithelial cells. Small central white circles are due to GFP in the lens that was visible through a pupil and masked corneal GFP. **A**: A time lapse sequence of central corneal images of a GFP-*Dstn^corn1^* mouse from six weeks to 42 weeks of age is shown. **B**: The same eye is shown as in the previous panel (**A**), but a temporal side view from 42 weeks to 79 weeks is shown. **C**: A time lapse sequence of central corneal images of another GFP-*Dstn^corn1^* mouse is shown. **D**: The same eye is shown at the same time points as in the latter panel (**C**) except that images were from a temporal side view. White curved lines are drawn to indicate a boundary between the cornea and the limbus in panels with an angled view. Bar: 1 mm.

To confirm this, cell movement was determined directly by in vivo time lapse fluorescence microscopy, tracking epithelial GFP patterns in 20 eyes with an average follow-up time of 39 weeks including four eyes over 52 weeks. Two of the representative sequences are shown in Figure 3. In the sequence of Figure 3A, epithelial GFP exhibited a mixture of diffuse and stripe patterns, suggesting that the direction of cell movements had not been established yet. At six weeks, radial stripes became the dominant pattern, indicating that there were general cell movements in a radial direction. By 10 weeks, however, GFP stripes turned into irregularly shaped patches, suggesting that the radial cell movements had stopped before 10 weeks. Thereafter, GFP patterns were diffuse and remained generally immobile while their size appeared to grow slowly in all directions (Figure 3A,C,D), which is perhaps reflecting gradual and random local cell movements, accompanied by clonal growth of GFP positive cells. A time lapse sequence in Figure 3B shows images up to 79 weeks and demonstrates that epithelial GFP remained generally stationary for the life of the animal as the natural life span of *Dstn^corn1^* and GFP-*Dstn^corn1^* mice is about 70-80 weeks (unpublished observations). This profile was in clear contrast to a normal cornea that exhibited constant centripetal movement of epithelial cells from the limbus to the corneal center [10].

### Cell division in a GFP-*Dstn^corn1^* cornea

**Figure 4:**
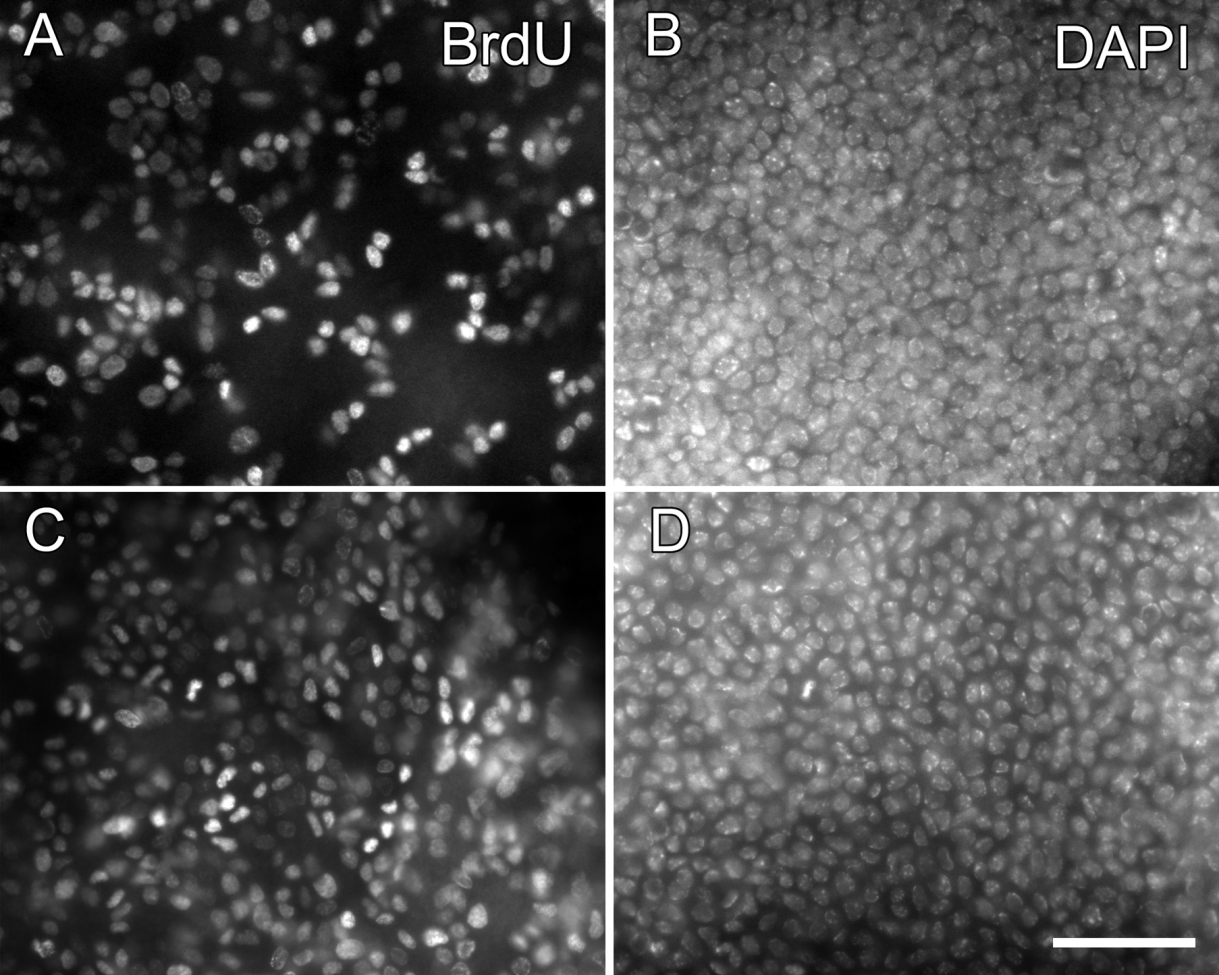
Distribution of mitotically active cells. DNA synthesis was  determined in a normal 18-week-old CAG-EGFP cornea (**A**,**B**) and a 20-week-old  GFP-Dstncorn1  cornea (**C**,**D**). Animals were labeled with systemic BrdU for 24 h continuously with an osmotic pump. BrdU positive cells were determined by immunohistochemistry with whole mount corneas and basal epithelial cells imaged (**A**,**C**). DAPI stain shows the entire population of basal epithelial cells of the same area (**B**,**D**). Bar: 50 µm.

Another parameter of epithelial homeostasis, cell division, was determined by two methods. In the first, we determined the rate of DNA synthesis during a 24 h period by labeling the animals with BrdU for 24 h straight using an osmotic pump implanted subcutaneously (Figure 4). Quantitation of the labeled cells indicated that more than 70% of basal epithelial cells in a GFP-*Dstn^corn1^* cornea incorporated BrdU during this period compared with 46% in a normal CAG-EGFP cornea (Table 1).

**Table 1:** BrdU-labeling rates in the basal epithelium of CAG-EGFP and GFP-*Dstn^corn1^* corneas.

	**BrdU-24 h (cells/mm^2^)**	**DAPI (cells/mm^2^)**	**BrdU-labeling rates (BrdU/DAPI)**
CAG-EGFP cornea (n=3, average 15 weeks)	6990±1000	15270±950	46%
GFP-*Dstn^corn1^* cornea (n=5, average 16 weeks)	10200±1000	14100±1420	72%

BrdU incorporation over a 24 h period was determined by continuous labeling with systemic BrdU. The numbers of DAPI-stained cells and BrdU positive cells in the basal epithelium were determined. The mean and the standard deviation per square millimeter are shown.

**Figure 5:**
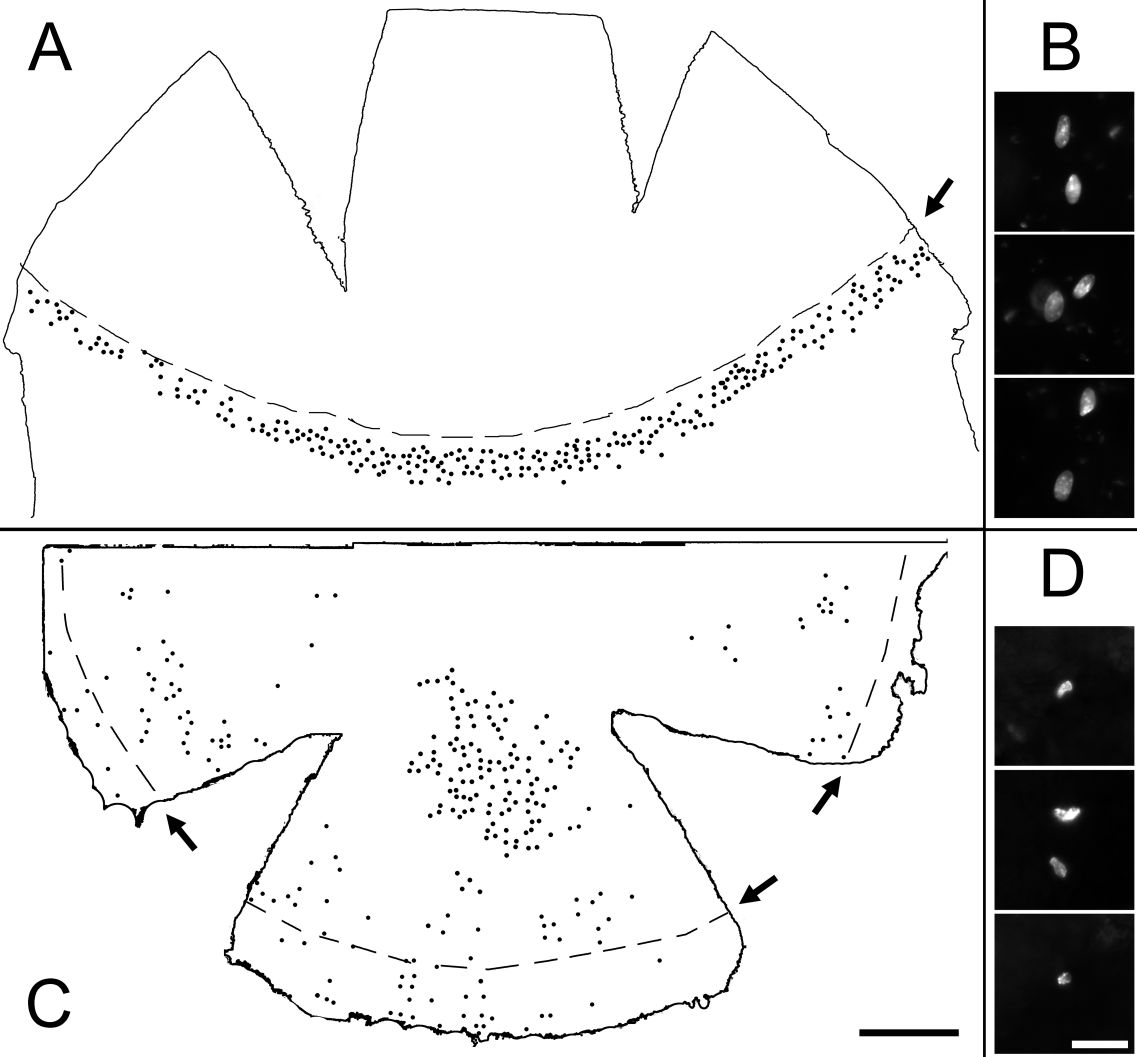
Distribution of corneal LRCs. LRCs were determined in a  20-week-old CAG-EGFP cornea (**A**,**B**) and a 28-week-old GFP-Dstncorn1 cornea  (**C**,**D**) by BrdU pulse chase labeling followed by BrdU immunohistochemistry. LRCs were determined by BrdU pulse chase labeling followed by BrdU immunohistochemistry. They were plotted manually under the microscope, surveying the entire inferior half of the cornea. Cuts were made to mount specimens flat either from the corneal side (**A**) or from the limbal side (**C**). An approximate position of a border between the cornea and the limbus is indicated by arrows and dashed lines. High power images of representative BrdU positive cells are also shown for a CAG-EGFP cornea (**B**) and a GFP-*Dstn^corn1^* cornea (**D**). **A** shows a representation of three CAG-EGFP corneas, and **C** shows a representative of eight GFP-*Dstn^corn1^* corneas that all showed similar results. Bar: 500 µm (**A**,**C**); 20 µm (**B**,**D**).

In the second method, we determined the distribution of label-retaining cells (LRCs) by a BrdU pulse chase, which was a two-week pulse label with BrdU with an osmotic pump and an eight-week chase during which nuclear BrdU was halved at each cell division. This protocol identified LRCs, which are regarded as a population of cells containing epithelial stem cells [12-14]. It has been well documented that normal corneas are devoid of such LRCs [13,15,16], and we confirmed this under our labeling protocol (Figure 5A). On the other hand, experiments with eight GFP-*Dstn^corn1^* corneas all showed that LRCs were widely distributed in the cornea (Figure 5C). Many of the BrdU positive nuclei were somewhat smaller and exhibited an irregular shape (Figure 5D) compared with oval-shaped limbal LRC nuclei of a CAG-EGFP eye (Figure 5B). In general, there were more LRCs in the peripheral cornea, mostly in the basal layer, than in the central portion where LRCs were mostly in layers just above the basal layer. Regardless of the distribution patterns, LRCs were clearly present in the cornea of GFP-*Dstn^corn1^* mice.

### Epithelial differentiation in the GFP-*Dstn^corn1^* cornea

**Figure 6:**
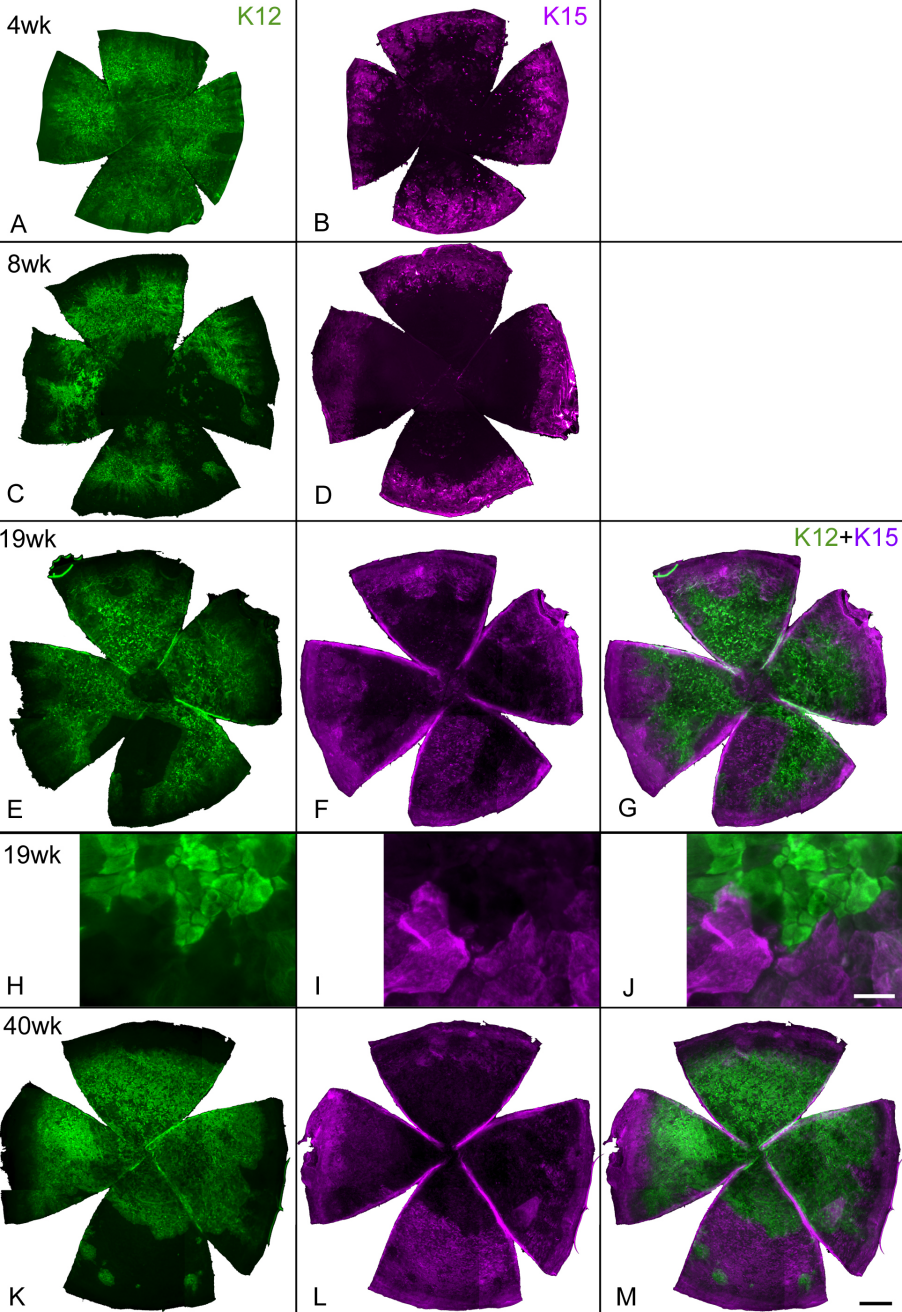
Expression of K12 and K15 in GFP-*Dstn^corn1^* corneas at different ages. Whole mounts of GFP-*Dstn^corn1^* cornea were used for immunofluorescence staining with K12 (**A**,**C**,**E**,**H**,**K** in green) and K15 (**B**,**D**,**F**,**I**,**L** in purple) at indicated ages. Only the cornea is shown. K12 patterns varied in different corneas, but they were present in wide areas of the GFP-*Dstn^corn1^* cornea at all ages examined. K15 staining was present in a wide peripheral zone of the cornea at four weeks (**B**), and the positive areas appeared to increase in older corneas (**D**,**F**,**L**) while it was only present at a very narrow peripheral zone of CAG-EGFP corneas adjacent to the limbus at all ages (not shown). **E** and **F**, **H** and **I**, and **K** and **L** are double staining of the same corneas, and corresponding composite images of K12 and K15 are also shown (**G**,**J**,**M**). **A**-**D** are all different corneas. In both a 19-week-old cornea (**E**-**G** for low power, **H**-**J** for high power) and a 40-week-old cornea (**K**-**M**), cellular patterns of K12 and K15 are mostly complementary and exclusive, although the match is not always perfect. Bars: 500 µm (**A**-**G**,**K**-**M**); 20 µm (**H**-**J**).

Results of cell migration and cell division indicated that epithelial cells of GFP-*Dstn^corn1^* cornea are not of the corneal phenotype. To determine whether GFP-*Dstn^corn1^* epithelial cells are of the conjunctival phenotype, we first examined keratin expression in GFP-*Dstn^corn1^* corneas using K12 as a corneal marker [17] and K15 as a conjunctival marker [18] (Figure 6). The epithelial surface of the GFP-*Dstn^corn1^* cornea was diverse, and no two corneas showed the same pattern of keratin expression. Nevertheless, there was a tendency that K12 positive cells were distributed in the central portion of the cornea while K15 positive cells were in the peripheral region in contact with the limbus. K12 and K15 patterns were nearly exclusive to each other in many parts of the cornea (Figure 6), although there were occasional cells that were positive with both or neither. Efforts to establish a correlation between histological changes and change of cell movements were not successful because histological patterns varied considerably among individual GFP-*Dstn^corn1^* corneas in the same age groups.

**Figure 7:**
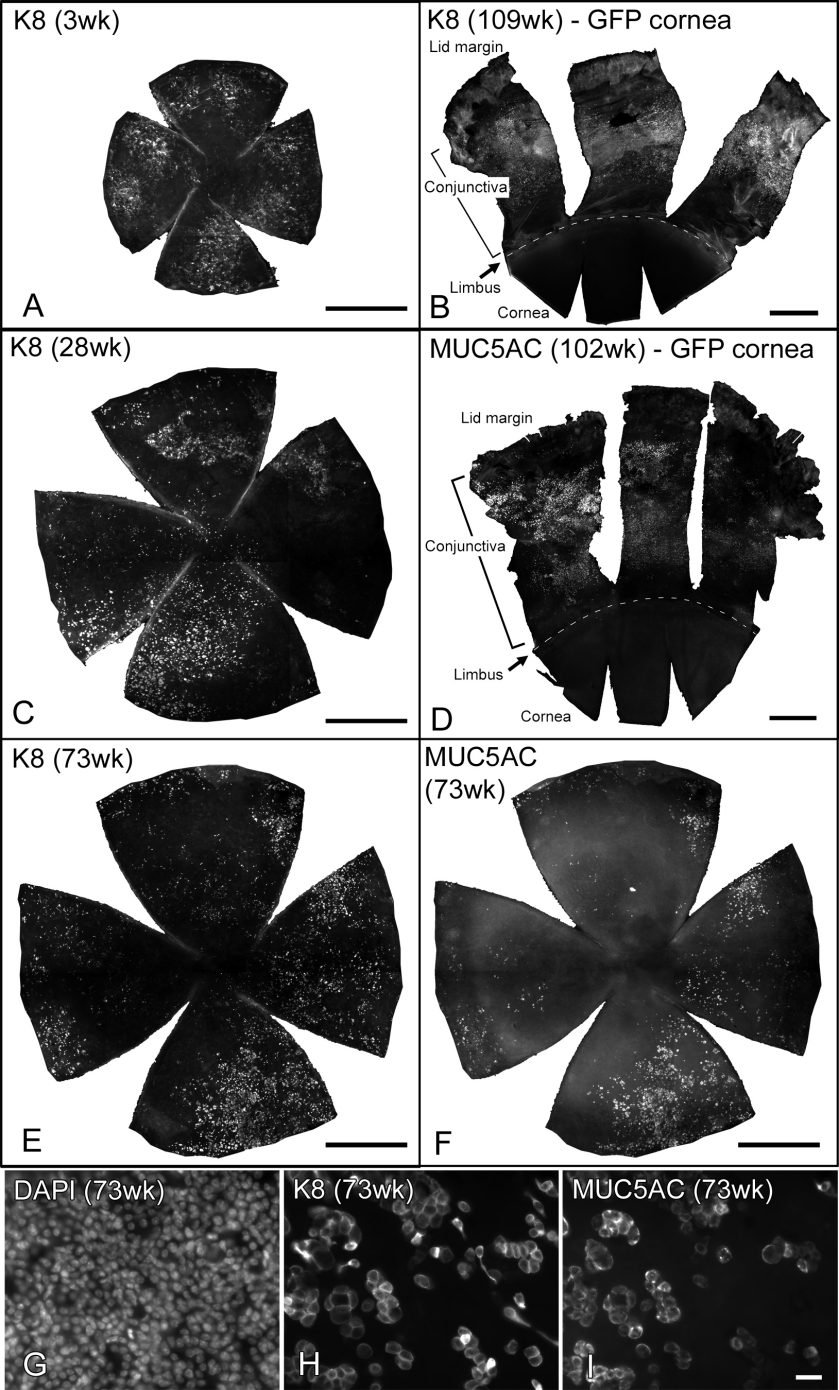
Expression of K8 and MUC5AC. Immunofluorescence staining with K8 (**A**-**C**,**E**,**H**) and MUC5AC (**D**,**F**,**I**) was performed with ocular surface whole mounts of GFP-*Dstn^corn1^* mice (**A**,**C**,**E**-**I**) and CAG-EGFP mice (**B**,**D**) at the indicated ages. For the GFP-*Dstn^corn1^* specimens, only the cornea is shown, and for the CAG-EGFP specimens, a superior half of the ocular surface containing cornea and conjunctiva up to the lid margin is shown together with an approximate position of the cornea-limbus boundary line shown as dashed lines. **B** and **D** show that K8 and MUC5AC are completely absent in normal CAG-EGFP corneas even at an advanced age while they are present in wide areas of the conjunctiva. **A**, **C**, and **E** show that K8 is widely distributed in GFP-*Dstn^corn1^* corneas at all ages. **E** and **F** show the same GFP-*Dstn^corn1^* cornea (73 weeks old) with double staining with K8 and MUC5AC, respectively. **G**, **H**, and **I** show high power images of the same 73-week-old cornea with triple staining with DAPI, K8, and MUC5AC. Most of the MUC5AC positive cells are also positive with K8. Bars: 500 µm (**A**-**F**), 20 µm (**G**-**I**).

We also looked at expression of K8 that is normally found in the conjunctiva [19,20] and the expression of a goblet cell marker, MUC5AC [21], neither of which was found in a normal cornea even at advanced ages (K8 in Figure 7B, MUC5AC in Figure 7D). K8 positive cells were present in all the GFP-*Dstn^corn1^* corneas we examined (Figure 7). They were scattered throughout the cornea with various patterns and formed a cluster of several cells but never a larger group. This K8 pattern was found as early as three weeks of age and appeared similar at all ages studied (Figures 7A,C,E). On the other hand, MUC5AC appeared to be absent in the cornea of GFP-*Dstn^corn1^* mice younger than 25 weeks (data not shown). However, a considerable number of MUC5AC positive cells were present in all five corneas that we examined at 51 weeks or older (51-73 weeks; Figure 7F). Double staining for K8 and MUC5AC revealed that nearly all MUC5AC positive cells were K8 positive (Figure 7E,F for low power; Figure 7H,I for high power).

## Discussion

Our results demonstrate that epithelial cells of a GFP-*Dstn^corn1^* mouse cornea are different from those of a normal cornea and that they are distinguished by the following unique characteristics; 1) they are generally immobile, 2) they exhibit higher rates of cell division, 3) some of them are LRCs, and 4) they show a mixture of corneal (K12) and conjunctival (K15, K8, and MUC5AC) phenotypes. These observations make it clear that epithelial homeostasis is abnormal in a GFP-*Dstn^corn1^* cornea.

### Epithelial homeostasis in a GFP-*Dstn^corn1^* cornea

One of the implications of our findings is that the epithelium of a GFP-*Dstn^corn1^* cornea may contain epithelial stem cells within it. This is suggested by the observation that epithelial cells were generally stationary during the life of the animal and yet mitotically active, indicating that the GFP-*Dstn^corn1^* epithelium is a self-sustained tissue, which would contain its own stem cells. This notion was supported by the finding that LRCs were present in the epithelium of a GFP-*Dstn^corn1^* cornea. Although all LRCs are clearly not stem cells [14], some LRCs are likely to be stem cells [12,13]. The situation is similar to the skin where cells are generally immobile in a lateral direction and maintained by keratinocyte stem cells within the skin [22]. This is, however, unlike a normal corneal epithelium, which is maintained by a constant influx of epithelial cells that are generated by limbal stem cells [23-25].

A comparison of epithelial homeostasis suggests that the epithelium of a GFP-*Dstn^corn1^* cornea resembles that of a conjunctiva or a conjunctivalized cornea. This point is based on our characterization of the normal conjunctival epithelium [8] and the epithelium of a conjunctivalized cornea (unpublished data), both of which are self-sufficient in cell renewal and appear to contain uniformly distributed stem cells. However, one marked difference is that a *Dstn^corn1^* cornea exhibits several patches of epithelial hyperplasia throughout the cornea while such hyperplastic zones do not usually exist in a conjunctival epithelium or a conjunctivalized cornea. One parameter that we have not measured is a rate of cell desquamation, which may also be a contributing factor in the hyperplastic phenotype.

### Epithelial differentiation in a GFP-*Dstn^corn1^* cornea

An adult GFP-*Dstn^corn1^* cornea contained a mixed population of cells expressing markers for corneal and conjunctival epithelium. MUC5AC-positive goblet cells were also present in older mice. Non-corneal cells may have migrated from the conjunctiva, but we failed to detect directed epithelial cell movements in a GFP-*Dstn^corn1^* cornea including those from the conjunctiva into the cornea for the entire life in some animals. Therefore, it is likely that those cells of a conjunctival phenotype in the cornea arose by differentiation of epithelial stem cells in situ rather than immigration of conjunctival cells into the cornea. This in turn suggests that a GFP-*Dstn^corn1^* cornea contains 1) three types of stem cells, each specific for corneal epithelial cells, conjunctival epithelial cells, or goblet cells; 2) multi-potent stem cells that are capable of differentiating into all three types of cells (and perhaps others); or 3) a combination of both. Further investigation may shed light on the nature of tissue-specific stem cells and their differentiation.

As to differentiation cues, variable patterns of K12 and K15 in GFP-*Dstn^corn1^* corneas of the same age groups suggest that there is a stochastic element to the epithelial differentiation, i.e., some epithelial cells go through abnormal differentiation (manifested by expression of odd keratins), but it is not possible a priori to predict which cells do and where in the cornea they appear. The stochasticity may be a feature of epithelial differentiation in a normal cornea that continues well into adulthood because it has been reported that K12 expression in the mouse cornea did not stabilize until the mouse was six months old [26].

### A destrin mutation

Our results with GFP-*Dstn^corn1^* mice demonstrate that destrin-defective epithelial cells lost the ability to move centripetally at a constant rate in the cornea. Given the involvement of cofilin/destrin in cell movement in a variety of tissues [3,27], it is likely that loss of destrin is directly related to the loss of epithelial cell movement in a GFP-*Dstn^corn1^* cornea. On the other hand, it is not clear whether the loss of destrin is the primary cause of other epithelial abnormalities such as increased division rates and hyperplasia. At this time, it is equally possible that such abnormalities are cumulative results of secondary events such as lack of cell movement triggered by destrin loss. Further investigations are required to determine how destrin deficiency leads to a variety of epithelial abnormalities at a molecular level.

### Epithelial movement and vascularization

Having established the abnormal cell movement in a *Dstn^corn1^* cornea in this study, we note an apparent correlation between epithelial cell movement and underlying stromal vascularization. On one hand, epithelial cells exhibit constant centripetal movement in a normal avascular cornea [10]. On the other hand, epithelial cells are generally stationary in a vascularized cornea including a *Dstn^corn1^* cornea (this study), a pathological conjunctivalized cornea [28], and a normal conjunctiva [8]. Similarly, epithelial cell movements were reportedly disturbed in a vascularized *Pax6*^+/−^ mouse cornea [29,30]. These observations raise an intriguing possibility that constant homeostatic epithelial cell movement may be a required component of avascularity in a normal cornea and that lack of such movement may trigger vascularization. Many variations of this scheme are possible including those that incorporate a vital role of sflt-1 [7] or VEGFR3 [6], but further discussion should await more experimental results. *Dstn^corn1^* mice should serve as a valuable tool for this purpose, especially in combination with *Dstn^corn1–2J^* [2], an independent destrin mutant mouse line with no corneal vascularization.
